# Surveillance of avian influenza virus type A in semi-scavenging ducks in Bangladesh

**DOI:** 10.1186/1746-6148-9-196

**Published:** 2013-10-07

**Authors:** Amina Khatun, Mohammed Giasuddin, Kazi Mehetazul Islam, Sazeda Khanom, Mohammed Abdus Samad, Mohammad Rafiqul Islam, Monira Noor, Jamal Uddin Bhuiyan, Won-Il Kim, Seong Kug Eo, Md Masudur Rahman

**Affiliations:** 1 College of Veterinary Medicine and Bio-Safety Research Institute, Chonbuk National University, Jeonju 561-756, Republic of Korea; 2 National Reference Laboratory for Avian Influenza, Bangladesh Livestock Research Institute, Savar, Dhaka, Bangladesh; 3 Faculty of Veterinary and Animal Sciences, Sylhet Agricultural University, Sylhet 3100, Bangladesh; 4 Department of Pathology, Faculty of Veterinary and Animal Sciences, Sylhet Agricultural University, Sylhet 3100, Bangladesh

**Keywords:** Avian influenza, Surveillance, Semi-scavenging ducks, Bangladesh

## Abstract

**Background:**

Ducks are the natural reservoir of influenza A virus and the central host for highly pathogenic avian influenza (H5N1), while domestic ducks rearing in semi-scavenging system could serve as re-assortment vessels for re-emerging new subtypes of influenza viruses between birds to human. Avian influenza virus (AIV) surveillance in Bangladesh has been passive, relying on poultry farmers to report suspected outbreaks of highly pathogenic H5N1 influenza. Here, the results of an active surveillance effort focusing on the semi-scavenging ducks are presented.

**Result:**

A total of 2100 cloacal swabs and 2100 sera were collected from semi-scavenging ducks from three wintering-sites of Bangladesh during three successive winter seasons, December through February in the years between 2009 and 2012. Virus isolation and identification were carried out from the cloacal swabs by virus propagation in embryonated hen eggs followed by amplification of viral RNA using Avian influenza virus (AIV) specific RT-PCR. The overall prevalence of avian influenza type A was 22.05% for swab samples and 39.76% ducks were sero-positive for avian influenza type A antibody. Extremely low sero-prevalence (0.09%) of AIV H5N1 was detected.

**Conclusions:**

Based on our surveillance results, we conclude that semi-scavenging ducks in Bangladesh might play important role in transmitting Avian Influenza virus (AIV) type A. However, the current risk of infection for humans from domestic ducks in Bangladesh is negligible. We believe that this relatively large dataset over three winters in Bangladesh might create a strong foundation for future studies of AIV prevalence, evolution, and ecology in wintering sites around the globe.

## Background

Influenza-virus is a negative-strand RNA virus belonging to the family Orthomyxoviridae and has been classified into subtypes by the surface proteins hemagglutinin (HA) and neuraminidase (NA). At present, sixteen HA subtypes and nine NA subtypes have been recognized [1]. Avian influenza viruses (AIVs) are further classified into two distinct groups, low pathogenic avian influenza (LPAI) viruses and highly pathogenic avian influenza (HPAI) viruses, based on their ability to produce clinical disease in chickens. All HPAI viruses that cause generalized fatal disease belong to either the H5 or H7 subtypes [2-4]. However, H5 and H7 viruses may circulate in the nature as LPAI strains for certain period of time and can mutate into HPAI strains mainly by antigenic drift or antigenic shift and LPAI viruses may also mutate to HPAI virus strains during infection in chickens [5]. The viruses are distributed worldwide and cause serious economic losses when outbreaks occur as clinical disease mostly in chickens, turkeys, and other gallinaceous birds. Moreover, the viruses have been isolated from a wide variety of animals, including humans, pigs, horses, tigers, cats, and other felids, ratites such as ostriches, emus, and rheas and sea mammals [6-10] and therefore, are of sparked concern now due to their fatality and zoonoses.

Migrating wild waterfowl are assumed to represent a risk for the transmission of infectious diseases to poultry [11]. Some of the AIV subtypes circulating in waterfowl may cause disease outbreaks when introduced into commercial poultry [12] with resulting serious economic losses, as occurred in the 1983–1984 outbreaks in Pennsylvania [13]. Virus representatives of all 16 HA and all 9 NA subtypes have been isolated from waterfowl [14]. Most avian influenza viruses replicate preferentially in the gastrointestinal tract of wild ducks, are excreted at high levels in feces, and are transmitted through the fecal-oral route [15]. Generally migratory waterfowl spread AIV without showing any clinical signs of disease [10].

**Figure 1:**
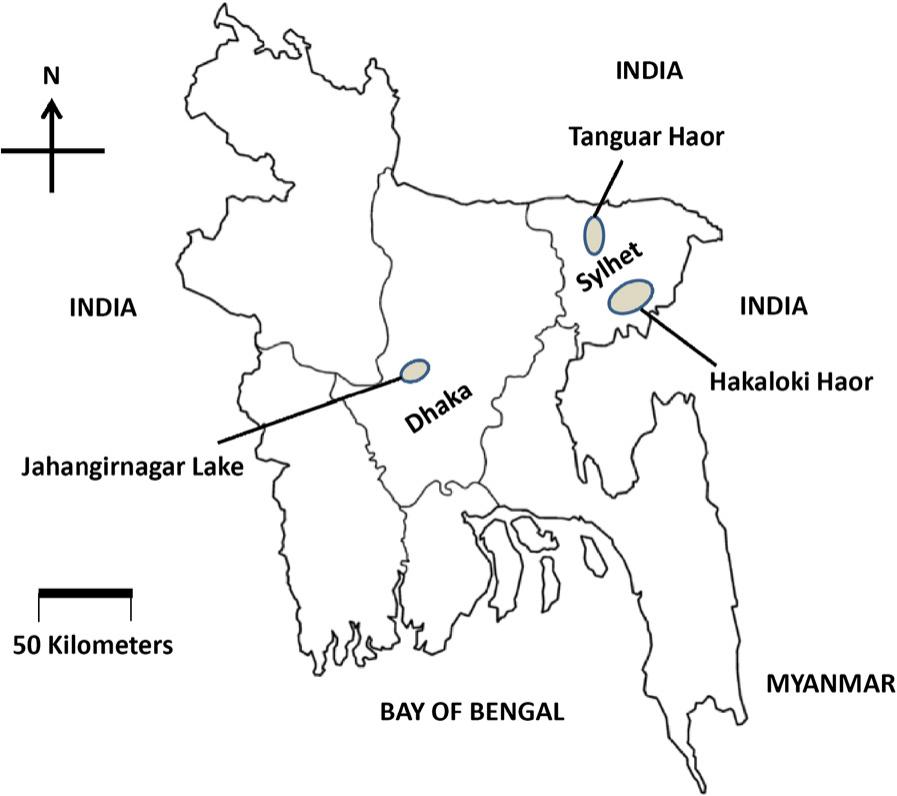
**Map of Bangladesh that summarizes the locations of sampling sites at three wintering sites.** A total of 4200 samples (2100 cloacal swabs and 2100 sera) were screened for influenza A by virus identification using RT-PCR and by specific antibody detection using iELISA respectively. The approximate locations of the sampling sites are presented on the map. Hakaluki and Tanguar Haors are two largest marsh wetland resources in Sylhet division and Jahangirnagar Lake is an important Lake in Dhaka division of Bangladesh. Lots of migratory birds visited these Haors and Lake during winter season. Both cloacal swabs and sera samples were collected from semi-scavenging ducks reared surrounding the water locked areas.

In Bangladesh, HPAI had been identified for the first time in March 2007 by National Reference Laboratory for Avian Influenza (NRL-AI) after passing a long immediate risk period which was reconfirmed by the International Reference Laboratory in UK and a regional laboratory in Thailand [16]. Human infections with HPAI H5N1 have been reported in Bangladesh and Myanmar (http://www.oie.int/eng/info_ev/en_AI_avianinfluenza.htm ). As of February24, 2011, there have been 384 reported outbreaks of HPAI H5N1 subtype at either backyard or commercial farms in 49 of the 64 districts of Bangladesh [17]. Bangladesh has a long border with India and Myanmar (Figure 1). Moreover, during the winter (from December through February), open water bodies in Bangladesh are shared by large number of migratory waterfowl and domestic semi-scavenging ducks. As a result, the domestic ducks might get AIVs from migratory waterfowls and might act as a natural reservoir of AIVs without showing clinical disease. In fact, Bangladesh, with duck stocks of 38.1 million, has the third largest duck population in the world [18]. Also, small scale commercial poultry farms with poor bio-security are widespread throughout the country in addition to household village chicken. Many households keep chickens and ducks on same premises [18] and domestic semi-scavenging ducks are often in close contact with poultry, livestock, and humans in the same property. Therefore, domestic ducks may play a major role in the ecology of AIVs in Bangladesh and may act as potential vessels for their genetic re-assortment [18] and thus demand active surveillance. Unfortunately, most of the information regarding influenza infection in Bangladesh has focused on passive surveillance of backyard or commercial farms [19,20], relying on poultry farmers to report suspected outbreaks of HPAI. Recently, an active surveillance for AIV on live bird markets of Bangladesh has been conducted and seven LPAI virus strains have been isolated with predominantly H9N2 strains and H5N1 strain has been observed at extremely low prevalence [21]. This study primarily details influenza infection in semi-scavenging ducks surrounding three important wintering-sites of Bangladesh because of the hypothesized role that the semi-scavenging domestic ducks play on the epidemiology of AIV.

## Results

### Isolation and identification of avian influenza virus type A

**Figure 2:**
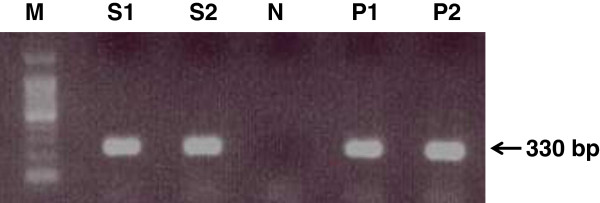
**Identification of AIV Type A by amplification of coding region of NP gene using RT-PCR.** Viral RNA extracted from HA positive samples (allantoic fluid) were employed to amplify coding region of NP (nucleoprotein**)** gene using specific primer pair. The arrow in agarose gel image indicates amplified NP gene (330 bp). Lanes M, size marker; S1 and S2, test samples; N, negative control; P1 and P2, positive control (NP gene as template).

In order to isolate and identify avian influenza type A, a total of 2100 cloacal swab samples were collected from semi-scavenzing domestic ducks of three different locations of Bangladesh and subjected to virus propagation by embryo inoculation, screening of HA activity and amplification of coding region of NP gene of avian influenza virus type A by RT-PCR. Out of 2100 samples, 1019 were positive for HA activity by the hemagglutination tests (Table 1). As NDV is ubiquitous in the world and ND live vaccines are used worldwide, the HI test with NDV specific antiserum was also conducted with HA positive samples to determine the NDV positive cases. All HA positive samples including NDV positive samples were tested for AIV identification by RT-PCR. NDV positive samples were included to determine co-infection of NDV and AIV. Out of 1019 HA positive samples, 505 were positive for NDV and 463 were positive for AIV type A (Table 1) as determined by ND HI test and RT-PCR respectively. RT-PCR results were validated by the detection of 330 bp sized amplicon of NP gene of AIV type A similar to positive control (Figure 2). A fragment of 330 bp NP gene of AIV type A was used as positive control in RT-PCR.

**Table 1:** Isolation and identification of AIV type A from cloacal swabs during the period of December’2009 to February’2012

**Winter season**	**Division**	**Wintering sites**	**Total no. of sample**	**No. of HA positive samples**	**No. of NDV positive samples**	**No. of AIV positive cases in NDV positive samples**	**RT-PCR positive samples of AIV**	**Prevalence of AIV (%)**	**Seasonal prevalence (%)**
	Sylhet	Hakaluki Haor	450	278	126	19 (15.05)^*^	146	32.44	26.89
Dec. 2009 to Feb. 2010		Tanguar Haor	225	94	52	6 (11.54)	51	22.67	
	Dhaka	Jahangirnagar Lake	225	73	37	4 (10.81)	45	20.00	
	Sylhet	Hakaluki Haor	300	145	75	7 (9.33)	63	21.00	18.50
Dec. 2010 to Feb. 2011		Tanguar Haor	150	64	39	3 (7.69)	27	18.00	
	Dhaka	Jahangirnagar Lake	150	58	23	2 (8.69)	21	14.00	
	Sylhet	Hakaluki Haor	300	163	75	5 (6.67)	58	19.33	18.33
Dec. 2011 to Feb. 2012		Tanguar Haor	150	71	37	2 (5.41)	23	15.33	
	Dhaka	Jahangirnagar Lake	150	73	41	2 (4.88)	29	19.33	
Total			**2100**	**1019**	**505**	**50 (9.90%)**	**463**	**22.05**	

A total of 2100 cloacal swab samples from semi-scavenging domestic ducks were tested for virus isolation through chicken embryo inoculation following by Haemagglutination test and identification was confirmed by RT-PCR from HA positive samples (allantoic fluid).

^*^Values in the parenthesis indicates per cent positive.

### Prevalence of avian influenza type A in semi-scavenging domestic ducks

The overall prevalence of AIV type A for three winter seasons was recorded as 22.05% and out of 505 NDV positive cases 50 (9.90%) were positive for AIV type A (Table 1). The seasonal prevalence of AIV type A from December 2009 through February 2010, from December 2010 through February 2011 and from December 2011 through February 2012 was 26.89%, 18.50% and 18.33% respectively. Like seasonal differences, wintering-site differences of AIV isolation existed which are shown in Table 1 but these differences are not statistically significant in both the cases. The virus isolation rate from December 2009 through February 2010 was highest from Hakaluki Haor (32.44%) followed by Tanguar Haor (22.67%) and Jahangirnagar Lake (20.00%). Similar trend in virus isolation rate among three wintering-sites existed in two successive winter seasons. It is mentioned here that we could not perform sub-typing of the isolates due to our limited facilities. However, we checked the status of sero-prevalence of AIV H5N1 in semi-scavenging ducks from all wintering sites and three successive winter seasons which was our major concern.

### Sero-prevalence of avian influenza in semi-scavenzing domestic ducks

A total of 2100 sera samples were tested by indirect ELISA to detect the specific antibodies for AIV type A and AIV H5N1. The overall sero-prevalence of AIV type A in three successive winter seasons was recorded as 39.76% (Table 2). The highest prevalence was recorded from December 2009 through February 2010 (43.89%) followed by two successive winters, i.e., from December 2010 through February 2011 (38.50%) and from December 2011 through February 2012 (34.83%). Like seasonal differences, wintering-site differences of sero-prevalence of AIV type A antibody existed which are shown in Table 2. The trend of sero-prevalence of AIV type A antibody in three different wintering-sites is same as virus isolation rate as shown in Table 2. Out of 835 sero-positive samples for AIV type A specific antibody, only two samples were positive for AIV H5N1 specific antibody with a prevalence rate of 0.09% which is negligible. These samples came from Hakaluki Haor and Tanguar Haor of Sylhet division respectively during the period from December 2009 through February 2010.

**Table 2:** Sero-prevalence of AIV type A and subtype H5N1 in semi-scavenging domestic ducks during the period from December’2009 to February’2012

**Winter season**	**Division**	**Wintering sites**	**Total no. of sample tested by iELISA**	**AIV type A positive sample**	**Sero-prevalence (%) of AIV type A**	**AIV H5N1 positive sample**	**Sero-prevalence (%) of AIV H5N1**	**Seasonal sero-prevalence (%) of AIV type A**
	Sylhet	Hakaluki Haor	450	213	47.33	01	0.22	43.89
Dec. 2009 to Feb. 2010		Tanguar Haor	225	95	42.22	01	0.44	
	Dhaka	Jahangirnagar Lake	225	87	38.67	00	00	
	Sylhet	Hakaluki Haor	300	121	40.33	00	00	38.50
Dec. 2010 to Feb. 2011		Tanguar Haor	150	58	38.67	00	00	
	Dhaka	Jahangirnagar Lake	150	52	34.67	00	00	
	Sylhet	Hakaluki Haor	300	113	37.67	00	00	34.83
Dec. 2011 to Feb. 2012		Tanguar Haor	150	53	35.33	00	00	
	Dhaka	Jahangirnagar Lake	150	43	28.67	00	00	
Total			**2100**	**835**	**39.76**	**02**	**0.09**	

A total of 2100 sera samples were tested to determine the antibody of AIV type A and subtype H5N1 by indirect ELISA.

## Discussion

Bangladesh is comprised of several large rivers and their tributaries which create a river delta covering nearly 90% of the country. Additionally, there are many haors, bills, lagoons, lakes and marshland in Bangladesh which make it well suited for duck rearing. Domestic ducks are often in close contact with poultry, livestock, and humans in the same premises in Bangladesh. Scavenging duck farming has been proposed as an important contributor to HPAI in poultry flocks in Southeast Asia, predominantly on the basis of findings obtained through spatial analyses of national surveillance data of HPAI outbreaks [22,23]. Assessment of HPAI movement across continents is of great concern to researchers working with AIV because of the panzootic HPAI with H5N1 viruses in Asia. Influenza surveillance in Bangladesh began in 2007 and was focused on the commercial and backyard farms only [22,23]. All these surveillance are mostly passive relying on the poultry farmers to report suspected outbreaks of HPAI. Here we concentrated our study on the active surveillance for AIV in semi-scavenging ducks to find out their role in AIV epidemiology. Results of our current study indicate that semi-scavenging ducks might be a source of infection for poultry in Bangladesh.

The data presented in this report are from cloacal swab and sera samples collected from semi-scavenging ducks from December 2009 through February 2010, from December 2010 through February 2011 and from December 2011 through February 2012 at three wintering sites in Sylhet and Dhaka division of Bangladesh. The overall AIV type A positive cases were recorded as 463 out of 2100 swab samples and the prevalence rate was 22.05% which is comparable to the recent prevalence report on live bird market in Bangladesh [21]. According to our results, semi-scavenging ducks are identified as a potential threat on AIV transmission in backyard and commercial poultry with low bio-security which is in agreement with the recent AI surveillance study in the West Bengal State of India, a neighboring country of Bangladesh [24]. The highest prevalence was recorded as 26.89% in the winter during December 2009 through February 2010 which may have relation with 384 reported outbreak of HP H5N1 subtype at either backyard or commercial farms in 49 of the 64 districts of Bangladesh in 2010 [17]. Although we did not perform sub typing of the isolates, it is assumed from the recent study on live bird market that new subtypes of AIV, with various combinations of hemagglutinins and neuraminidase, are currently circulating [21].

With respect to wintering sites, highest prevalence was reported from swab collected surrounding the Hakaluki haor at three successive winters is the largest marsh wetland ecological system of Eastern Bangladesh and one of Asia's larger marsh wetland resources wherein millions of migratory waterfowls harbor during winter season. Therefore, it is possible that larger number of semi-scavenging ducks get infected from migratory waterfowls during sharing the same water bodies. We got higher sero-prevalence rate (39.76%) of AIV type A than that from swab samples which may be due to clearance of active infection, however, antibody persist long time. Normally AIVs persist up to 4 wk in individual ducks [10] and AIV will be persisted longer if new susceptible birds join the flock. We detected specific antibody for AIV H5N1 in two sera samples out of 835 sera samples positive for AIV type A specific antibody throughout the study period, and the overall prevalence was 0.09% which is nearly similar to the recent AIV H5N1 prevalence report (0.08%) on live bird market in Bangladesh [19]. Bangladesh first experienced HPAI (H5N1) in early 2007 followed by 2008 and 2009 with highest peak during the period of January to May 2008 [16]. After the outbreak of HPAI in early 2007, around 1.69 million birds were culled and disposed off and 2.25 million eggs were destroyed concurrently. Additionally, strick biosecurity was imposed along with surveillance and monitoring. This might be the reason of getting negligible sero-prevalence of AIV H5N1 in our study. Although we could not screened the sero-positive samples for other subtypes, it is possible that there might have several LPAI strains in our isolates and HPAI strains might be evolved from such LPAI strains which is alarming for both poultry and human health in Bangladesh.

## Conclusions

We concluded here that the present surveillance study on semi-scavenging domestic ducks for Avian influenza virus (AIV) in Bangladesh increases our understanding on the ecology and epidemiology of AIVs. Knowledge of circulating AIVs might help the researchers in designing suitable measures to prevent introduction and spread of Avian influenza viruses (AIVs) in poultry. We believe that this relatively large dataset over three winters in Bangladesh might create a strong foundation for future studies of AIVs prevalence, evolution, and ecology in wintering sites around the globe.

## Methods

### Sampling location and sample collection

Active avian influenza surveillance was carried out in semi-scavenging domestic ducks in Bangladesh during winter seasons from December through February 2009–2012. Two divisions of Bangladesh, namely Sylhet and Dhaka, were selected for sampling where there have natural lakes and haors (haor means a marsh wetland ecological system). Hakaluki Haor and Tanguar Haor both located in Sylhet division and Jahangirnagar Lake located in Dhaka division are three important wintering sites where lots of migratory waterfowls from Siberia visit in mid November and stay up to February. Semi-scavenging domestic ducks also share these water bodies with migratory birds. A total of 2100 cloacal swab and 2100 sera samples were collected from the semi-scavenging domestic ducks surrounding three wintering sites which are depicted in Figure 1. Two types of sample, cloacal swab and blood for sera, were collected from each duck. Distribution of samples according to seasons and wintering sites are shown in Table 3. Cloacal swabs were collected randomly in sterile tubes containing virus transport media (VTM) (MART; Remel, Inc., Lenexa, KS, USA) for virus isolation and identification. Blood samples were collected from corresponding ducks in sterile syringe for serum separation to study sero-prevalence. None of the ducks that were sampled or observed near the sampling location exhibited signs of disease, and none of the sampling sites were reported as an outbreak area during the study. After collection, all samples were kept at 4°C and transported to the National Reference Laboratory for Avian Influenza (NRL-AI), Bangladesh Livestock Research Institute (BLRI), Savar, Dhaka within 2 days maintaining proper cooling chain. The samples were stored at −20°C until tested. All experimental procedures and animal management procedures were undertaken in accordance with the requirements of the Animal Care and Ethics Committees of BLRI. The animal facility of BLRI is fully accredited by the National Association of Laboratory Animal Care.

**Table 3:** Wintering sites and distribution of samples collected from semi-scavenging domestic ducks between December 2009 to February’ 2012

**Division**	**Wintering site**	**Time scale of sample collection**																		**Total no. of samples**	
		**Dec. 2009**		**Jan. 2010**		**Feb. 2010**		**Dec. 2010**		**Jan. 2011**		**Feb. 2011**		**Dec. 2011**		**Jan. 2012**		**Feb. 2012**		**Swab**	**Sera**
		*Swab	**Sera**	**Swab**	**Sera**	**Swab**	**Sera**	**Swab**	**Sera**	**Swab**	**Sera**	**Swab**	**Sera**	**Swab**	**Sera**	**Swab**	**Sera**	**Swab**	**Sera**		
Sylhet	Hakaloki Haor	150	150	150	150	150	150	100	100	100	100	100	100	100	100	100	100	100	100	1050	1050
	Tanguar Haor	75	75	75	75	75	75	50	50	50	50	50	50	50	50	50	50	50	50	525	525
Dhaka	Jahangirnagar Lake	75	75	75	75	75	75	50	50	50	50	50	50	50	50	50	50	50	50	525	525
Total no. of samples		300	300	300	300	300	300	200	200	200	200	200	200	200	200	200	200	200	200	2100	2100

A total of 4200 samples (2100 cloacal swabs and 2100 sera) were collected randomly from semi-scavenging domestic ducks reared surroundings the wintering sites through three successive winter seasons in Bangladesh.

^*^Swab means cloacal swab. Two samples (swab & blood for sera) were collected from each randomly selected duck.

### Virus isolation by embryo inoculation

Virus isolation was carried out from cloacal swabs through embryo inoculation of 10-days old embryonated chicken eggs. Prior to inoculation, cloacal swab samples in VTM were thawed completely, vortex, centrifuged at 12,000 × g for 3 minutes at 4°C. The supernatants were supplemented with penicillin G (final concentration of 1000 U/ml), streptomycin (1 mg/ml), gentamycin (100 μg/ml) and amphotericin B (10 μg/ml) and inoculated into the allantoic cavity of 10-day-old embryonating chicken eggs (three eggs per samples) at the dose rate of 200 μl/egg. PBS was inoculated into 5 eggs as negative control per batch. The eggs were incubated at 37°C for 72 hrs and candled twice daily to check the viability of the embryo. At the end of the incubation period or upon embryo death, the allantoic fluid was harvested and centrifuged at 12,000 × g for 3 min and tested by the hemagglutination test with 0.5% chicken red blood cells [25]. The samples that did not show hemagglutination activity after a second egg passage were considered negative for virus. Samples with hemagglutination activity were screened by hemagglutination inhibition (HI) test using Newcastle disease virus (NDV) specific antiserum [25] to determine the number of NDV positive samples.

### Identification of avian influenza virus type A by reverse transcription polymerase chain reaction (RT-PCR)

All HA positive samples including NDV positive samples were subjected to RT-PCR analysis to identify avian influenza virus type A. NDV positive samples were considered to determine mixed infection (NDV and AIV type A). Viral RNA was extracted from all HA positive samples (allantoic fluid) using QIAGEN RNeasy Mini Kit (Germany) according to the manufacturer’s instruction and subjected to reverse transcription polymerase chain reaction (RT-PCR) using QIAGEN One-Step RT-PCR Kit (Germany) and primer pair specific for nucleoprotein (NP) gene of avian influenza type A (Table 4) according to manufacturer’s instruction in order to amplify the coding region of nucleoprotein (NP) gene of avian influenza type A. Briefly, a reaction mixture of 25 μl volume contains 5.0 μl of 5× QIAGEN OneStep RT-PCR buffer, 1.0 μl dNTP Mix (10 mM of each dNTP), 5.0 μl of 5× Q-Solution, 1.0 μl of each primer (20 pmol each), 1.0 μl of QIAGEN OneStep RT-PCR Enzyme Mix, 0.25 μl of RiboLock™ RNase inhibitor (40 U/ μl) (Thermo Fisher Scientific Inc., Germany), 2.0 μl of RNA template ( 2 ng), and 8.5 μl of nuclease free water. The PCR condition for the amplification of NP gene was 50°C for 30 minutes (reverse transcription), initial denaturation at 95°C for 15 minutes, then PCR (35 cycles): denaturation at 94°C for 30 seconds, annealing at 55°C for 40 seconds, elongation at 72°C for 1 minute, followed by 72°C for 10 min (final extension) and holding at 4°C until collection. Amplicons were subjected to 1% agarose gel electrophoresis and visualized with ethidium bromide by ultraviolet light using an image documentation system.

**Table 4:** Primers for RT-PCR amplification of nucleoprotein (NP) gene of avian influenza type A virus

**Target gene**	**Primer ID**	*Primer sequences (5′-3′)	**Amplicon size (bp)**	**Reference**
Nucleoprotein (NP)	NP-1200 (Forward)	CAG **R**TA CTG GGC **H**AT AAG **R**AC	330	[21]
	NP-1529 (Reverse)	GCA TTG TCT CCG AAG AAA TAAG		

* Inclusions of degenerate nucleotides are indicated in bold. Codes for mixed bases position: **R** = A/G, **H** = A/T/C.

### Indirect Enzyme Linked Immunosorbent Assay (iELISA) for the detection of specific antibody against AIV type A and subtype H5N1

In order to detect specific antibody for AIV type A and subtype H5N1, indirect enzyme linked immunosorbent assay (iELISA) was performed with collected sera using commercially available pre-coated plates and pre-diluted ready to use reagents and buffer (IDEXX influenza A Ab Test, IDEXX AI H5 Ab Test, IDEXX Laboratories, Inc., Westbrook, Maine 04092, USA) according to the manufacturer’s instruction. Briefly, test sera were diluted in specific sample diluents to avoid nonspecific binding to the antigen-coated wells. Following incubation with serum unbound materials were washed away and the antigen-antibody complexes were detected with an anti-chicken immunoglobulin-enzyme conjugate. Detection of a serologic response in the test serum is demonstrated by conversion of chromogen by specific enzyme using a Spectra MAX340 automated ELISA reader (Molecular Devices, Sunnyvale, CA). Results were analyzed by calculation of an adjusted sample absorbance divided by the adjusted positive control absorbance (S/P ratio).

## Abbreviations

AIV: Avian influenza virus; BLRI: Bangladesh Livestock Research Institute; bp: Base pair of nucleotide sequences; HA: Hemagglutinin; HI: Hemagglutination Inhibition; HPAI: Highly Pathogenic Avian Influenza; iELISA: Indirect Enzyme Linked Immunosorbent Assay; LPAI: Low Pathogenic Avian Influenza; NA: Neuraminidase; NRL-AI: National Reference Laboratory for Avian Influenza; ND: Newcastle Disease; NDV: Newcastle Disease Virus; NP: Nucleo-Protein; RT-PCR: Reverse Transcription Polymerase Chain Reaction; VTM: Virus Transport Media.

## Competing interests

The authors declare that they have no competing interests.

## Authors’ contributions

AK: contributed during study design, collected and processed the samples, evaluated the data and helped in writing the manuscript; KMI, SK, NM and JUB: contributed to collect and process the samples; MAS and MRI: performed RT-PCR and ELISA; WIK and SKE: contributed to the study design and critically involved in writing the manuscript; MG and MMR: obtained the funding, contributed to the study design, evaluated the data and wrote the manuscript. All authors read and approved the final manuscript.
